# Caregiver-Reported Barriers and Facilitators to Deprescribing in Older Adults With Dementia: A Systematic Review

**DOI:** 10.1093/geroni/igaf122.992

**Published:** 2025-12-31

**Authors:** Tara Klinedinst, Sarah Joseph, Te-Lien Ku, April Schweikhard, Beth Fields, Lee Jennings

**Affiliations:** The University of Oklahoma Health Sciences Center, Oklahoma City, Oklahoma, United States; The University of Oklahoma Health Sciences Center, Oklahoma City, Oklahoma, United States; University of Wisconsin-Madison, Madison, Wisconsin, United States; The University of Oklahoma, Norman, Oklahoma, United States; University of Wisconsin–Madison, Madison, Wisconsin, United States; University of Oklahoma Health Sciences, Oklahoma City, Oklahoma, United States

## Abstract

Many older adults with ADRD are at heightened risk for inappropriate medication prescription, due to the complexity of managing dementia in addition to comorbid medical conditions. Deprescribing medications that are harmful or no longer beneficial can prevent illness and injury, and caregivers play a crucial role in this process. Unfortunately, guidance on decision-making and practical supports for caregivers interested in deprescribing is limited. Previous low-touch interventions have not significantly reduced prescriptions, indicating that older adults with ADRD and their caregivers may need more intensive supports and resources during the deprescribing process. To address this gap, we used systematic review methodology to characterize barriers and facilitators to deprescribing as reported by caregivers of older adults with ADRD. We used thematic analysis to synthesize qualitative study results (n = 8 articles). Barriers to deprescribing included challenges such as limited caregiver knowledge and skills, reliance on medications to regulate behavior, fear of “giving up” on treatment, and lack of collaborative relationship with providers. Facilitators included a trusting alliance with providers and proactive communication (framing deprescribing as “routine”); worsening of care recipient health and negative effects of medication also triggered deprescribing conversations. Findings suggest that caregiver engagement and confidence in deprescribing is influenced by multiple factors, with communication and trust playing a central role. Addressing caregiver hesitancy through improved provider communication, clearer deprescribing guidelines, and targeted education may enhance confidence and willingness to deprescribe. Future initiatives should prioritize incorporating caregiver perspectives into deprescribing strategies to enhance care recipient outcomes and support informed medication management.

